# Grill Workers Exposure to Polycyclic Aromatic Hydrocarbons: Levels and Excretion Profiles of the Urinary Biomarkers

**DOI:** 10.3390/ijerph18010230

**Published:** 2020-12-30

**Authors:** Marta Oliveira, Sílvia Capelas, Cristina Delerue-Matos, Simone Morais

**Affiliations:** 1 REQUIMTE-LAQV, Instituto Superior de Engenharia, Instituto Politécnico do Porto, R. Dr. António Bernardino de Almeida 431, 4200-072 Porto, Portugal; marta.oliveira@graq.isep.ipp.pt (M.O.); silvia30_andreia@sapo.pt (S.C.); cmm@isep.ipp.pt (C.D.-M.); 2 Ibero Massa Florestal, S.A., 3720-584 Oliveira de Azeméis, Portugal

**Keywords:** biomarkers of exposure, grill workers, total internal dose, monohydroxyl-PAHs (OH-PAHs), polycyclic aromatic hydrocarbons

## Abstract

Grilling activities release large amounts of hazardous pollutants, but information on restaurant grill workers’ exposure to polycyclic aromatic hydrocarbons (PAHs) is almost inexistent. This study assessed the impact of grilling emissions on total workers’ exposure to PAHs by evaluating the concentrations of six urinary biomarkers of exposure (OHPAHs): naphthalene, acenaphthene, fluorene, phenanthrene, pyrene, and benzo(a)pyrene. Individual levels and excretion profiles of urinary OHPAHs were determined during working and nonworking periods. Urinary OHPAHs were quantified by high-performance liquid-chromatography with fluorescence detection. Levels of total OHPAHs (∑OHPAHs) were significantly increased (about nine times; *p* ≤ 0.001) during working comparatively with nonworking days. Urinary 1-hydroxynaphthalene + 1-hydroxyacenapthene and 2-hydroxyfluorene presented the highest increments (ca. 23- and 6-fold increase, respectively), followed by 1-hydroxyphenanthrene (ca. 2.3 times) and 1-hydroxypyrene (ca. 1.8 times). Additionally, 1-hydroxypyrene levels were higher than the benchmark, 0.5 µmol/mol creatinine, in 5% of exposed workers. Moreover, 3-hydroxybenzo(a)pyrene, biomarker of exposure to carcinogenic PAHs, was detected in 13% of exposed workers. Individual excretion profiles showed a cumulative increase in ∑OHPAHs during consecutive working days. A principal component analysis model partially discriminated workers’ exposure during working and nonworking periods showing the impact of grilling activities. Urinary OHPAHs were increased in grill workers during working days.

## 1. Introduction

Kitchen and grillroom workers are daily exposed to several airborne gaseous and particulate pollutants formed during the preparation of foods using different cooking methods (e.g., frying, grilling, and roasting). Cooking fumes are thermal oxidative decomposition products containing several hazardous pollutants such as respirable particulate matter (PM), heavy metals, black carbon, heterocyclic amines, and volatile organic compounds including polycyclic aromatic hydrocarbons (PAHs), aldehydes, and carbonyls [1,2,3,4,5,6,7,8]. In 2010, the International Agency for Research in Cancer (IARC) included the emissions from high-temperature frying in the list of probable carcinogens to humans [9]. The released cooking fumes containing health-relevant pollutants will be absorbed (via inhalation and dermal contact) into human body of exposed workers and long-term exposures have been associated with potential health risks [6,10]. Some authors reported that restaurant workers have an increased risk of suffering from myocardial infarction, principally in cooks and kitchen’ workers [11,12,13]. It is known that cooking oil fumes may induce lipid peroxidation and the expression of different cytokines, which causes oxidative DNA damage in the epithelial cells of human lungs [4,5,14,15]. Other authors also reported a direct association between the exposure to the emissions of cooking activities with a higher risk to develop cancer in the respiratory tract of bakers and chefs among other food service workers [16,17].

PAHs are a group of more than a hundred different aromatic compounds that are produced during the (incomplete) combustion processes of carbonaceous materials. Some PAHs present toxic, mutagenic, and carcinogenic properties, being also known as endocrine disruptors [18,19,20,21]. PAHs released during grilling activities originated from the incomplete combustion of charcoal on the food surface, the pyrolysis of fat, protein, and carbohydrates at high temperatures, and with the direct contact of deeply heated dripping lipids over the flame [22]. PAHs with two and three aromatic rings (e.g., naphthalene, acenaphthylene, acenaphthene, fluorene, and phenanthrene) are the predominant compounds released during cooking and/or barbecuing activities [3,7,23]. Once absorbed into the human body, PAHs are biotransformed through complex metabolic and enzymatic mechanisms by enzymes of the P450 complex, being metabolized into different reactive intermediates that are further eliminated in the urine as monohydroxyl metabolites conjugated with glucuronides and sulfates [24]. Thus, the quantification of PAHs biomarkers of exposure, urinary deconjugated monohydroxyl metabolites (OHPAHs), allows assessment of each individual internal PAH exposure. Urinary 1-hydroxypyrene (1OHPy), the major metabolite of pyrene, and monohydroxyl-naphthalenes, -fluorenes, and -phenanthrenes are the most widely characterized PAH biomarkers of exposure in human biomonitoring studies [24]. Urinary 3-hydroxybenzo(a)pyrene (3OHBaP) is a well-known biomarker of exposure to carcinogenic PAHs [24,25]. Increased levels of urinary OHPAHs in occupationally exposed groups have been associated with a higher prevalence of early markers of inflammation processes, cardiovascular disease, oxidative stress, and DNA damage [26,27,28].

Several authors already proved the occupational exposure to PAHs in cooks, grill workers, and other kitchen staff as well as in street food vendors [1,2,7,8,10,15,29,30,31,32,33,34,35,36,37]. From those studies, only a few included workers’ biomonitoring [4,5,7,8,15,29,35,36,37]. Available data come principally from Asian (Chinese and Thai) workers; only one study was found from another geographical origin (Portugal) [35]. In addition, only three studies included the quantification of other monohydroxyl metabolites besides 1OHPy [7,8,35].

The concentrations of urinary biomarkers of exposure to six different PAHs, namely, naphthalene, acenaphthene, fluorene, phenanthrene, pyrene, and benzo(a)pyrene, was for the first time assessed in European grill workers. Individual levels and excretion profiles of the characterized urinary OHPAHs were determined during a regular working period and in the following resting days to evaluate the contribution of grilling emissions on grill workers’ total exposure to PAHs.

## 2. Materials and Methods

### 2.1. Study Population and Sampling Campaigns

**Figure 1:**
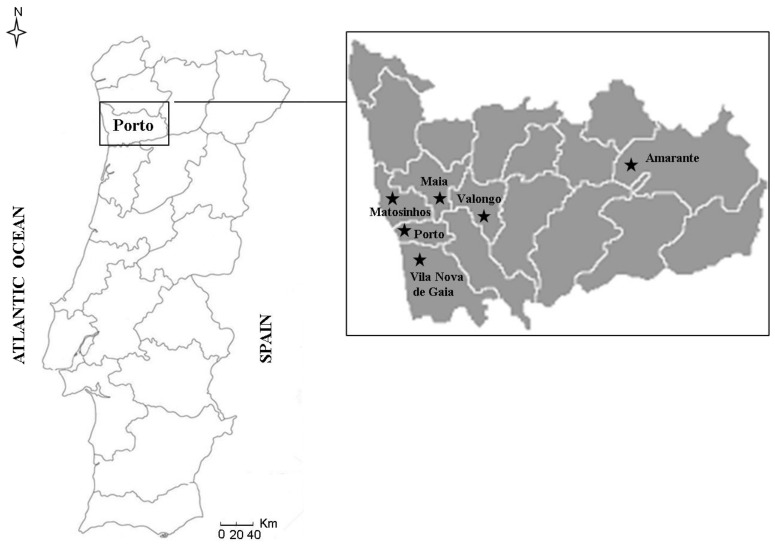
Geographical location of barbecue restaurants within the Oporto district.

All grill workers (Table 1) from six restaurants located in six cities from Oporto district, Northern Portugal (Figure 1), were invited to collaborate in this work. A regular workday was divided in two shifts, being the first between 10:00 a.m. and 2:00 p.m. and the second from 6:00 p.m. to 10:00 p.m.; depending on the workflow at the grillroom and/or the number of clients at the restaurant, subjects could work up to four more hours per day. Grill workers were responsible for all the grilling tasks, namely, the ignition of charcoal at the beginning of each working day, the preparation, maintenance and cleaning of grillroom, cooking the requested foods (mainly different types of meats and/or fishes), and the extinction of charcoal combustion.

All participants filled a questionnaire adapted from a World Health Organization questionnaire [38] and previously validated by the Ethics Committee of the University of Porto (CEUP). This work was performed according with The Code of Ethics of the World Medical Association (Declaration of Helsinki). Information on biometric data (age, weigh, and height), smoking habits and exposure to environmental tobacco smoke, duration of a regular working period and weekly days-off, diagnosed respiratory diseases, dietary habits (consumption of boiled, roasted, and grilled foods), and medication intake over the 7 days before the sampling campaigns was collected through the personal questionnaire. The inclusion criteria for participants in the study were: (i) exclusive grilling activities, i.e., subjects were not involved in other tasks at the restaurant; (ii) at least 2 years working as grill worker; and (iii) be a nonsmoker for at least 1 year. An informed consent form previously approved by CEUP (53/CEUP/2018) was signed by each grill worker.

Each participant collected a spot urine sample in a sterilized container at the end of the working day over a complete working week, including the resting days. After collection, urine samples were coded and frozen at −20 °C.

A researcher was daily present at the grillroom area to keep a record on other potential sources of exposure to PAHs.

### 2.2. Quantification of OHPAHs

Urinary 1-hydroxynaphthalene and 1-hydroxyacenaphthene (1OHNaph + 1OHAce), 2-hydroxyfluorene (2OHFlu), 1-hydroxyphenanthrene (1OHPhen), 1OHPy, and 3OHBaP were extracted according to Oliveira et al. (2016). Briefly, 10 mL of urine was buffered with acetate buffer (pH 5.0) and hydrolyzed with 80 µL of ß-glucuronidase/arylsulfatase from Helix pomatia (EC3.2.1.31/EC3.1.6.1; 5.5/2.6 U/mL; Roche Diagnostics, Indianapolis, USA) for 120 min at 37 °C (Binder KBWF, Tuttlingen, German). After hydrolysis, samples were loaded into Sep-Pak^®^Light Plus C18 (Waters; Sigma-Aldrich, Steinheim, Germany) that were preconditioned with 5.0 mL of methanol and 10.0 mL of water. After elution of samples, cartridges were cleaned with 10.0 mL of water and 10.0 mL of methanol/water (20:80; *v*/*v*). Cartridges were then completely dried, eluted with 20.0 mL of methanol/ethyl acetate (10:90; *v*/*v*), and evaporated till dryness (Büchi R200 rotavapor and a Büchi Vac V-500 pump). Extracts were redissolved in 300 µL of methanol. Urine extracts were analyzed in a C18 column (CC 150/4 Nucleosil 100–5 C18 PAH; Macherey–Nagel, Duren, Germany) by liquid chromatography (Shimadzu LC system, Shimadzu Corporation, Kyoto, Japan) with a fluorescence detection system. Detailed information related with the chromatographic characteristics can be found in Oliveira et al. [39] and Figure S1 of the Supplementary Materials presents representative chromatograms.

Calibrations using at least 6 calibration points of all the OHPAHs were performed in methanol (R^2^ ≥ 0.999) (Table S1 of the Supplementary Materials). The achieved limits of detection (LOD) varied from 0.80 (for 2OHFlu) to 0.195 μg/L urine (for 1OHNaph + 1OHAce), while the respective limits of quantification ranged between 2.80 and 0.650 μg/L urine.

Urinary levels of OHPAHs were corrected with the levels of urinary creatinine (µmol OHPAH/mol creatinine), which was determined according to the Jaffe colorimetric method [40]. The determination of urinary OHPAHs and creatinine was performed in triplicate.

### 2.3. Statistical Analysis

Data treatment was performed using SPSS (IBM SPSS Statistics 20) Statistica software (v. 7, StatSoft Inc., Tulsa, OK, USA). Levels of urinary OHPAHs were expressed as median, percentiles 25–75, and range. Since normality was not observed, urinary levels of OHPAHs were compared with nonparametric tests; a significant level of 5% was considered. When a PAH metabolite was not detected in the urine sample, the value of its respective LOD/√2 was considered [41]. Spearman correlation coefficients were calculated to evaluate the inter-relation between the individual and total OHPAHs (∑OHPAHs) levels in each grill worker.

## 3. Results

### 3.1. Characterization of Participants

Age of healthy and nonsmoking grill workers ranged between 20 and 48 years (mean of 35.7 years) and presented a mean body mass index (BMI) of 28.3 kg/m^2^ (23.7–35.6 kg/m^2^) (Table 1). A total of 67% of the participants were classified as overweight by presenting a BMI above 25 kg/m^2^ [42]. A regular work week was made of 5 consecutive working days followed by up to 2 days off, with grill workers spending a mean time of 9.6 ± 2.8 h/day at the grillroom area (Table 1). During the working period, the grillroom’ ventilation system was always tuned on.

### 3.2. Concentrations of Urinary Biomarkers

Levels of urinary OHPAHs were normalized with the individual concentrations of creatinine, to minimize the influence of diuresis and the variability caused by personal characteristics (e.g., fluids ingestion, practice of physical exercise, and body temperature). Concentrations of creatinine ranged from 0.432 to 2.90 g/L in the urine of grill workers, being these values within the acceptable range of values (0.3–3.0 g/L) defined by WHO [43]. The biomarkers 1OHPhen and 1OHPy were found in all the urines (100% detection rates) while 1OHNpah + 1OHAce and 2OHFlu were detected in 92–97% and 58–92% of the grill workers’ samples, respectively.

**Figure 2:**
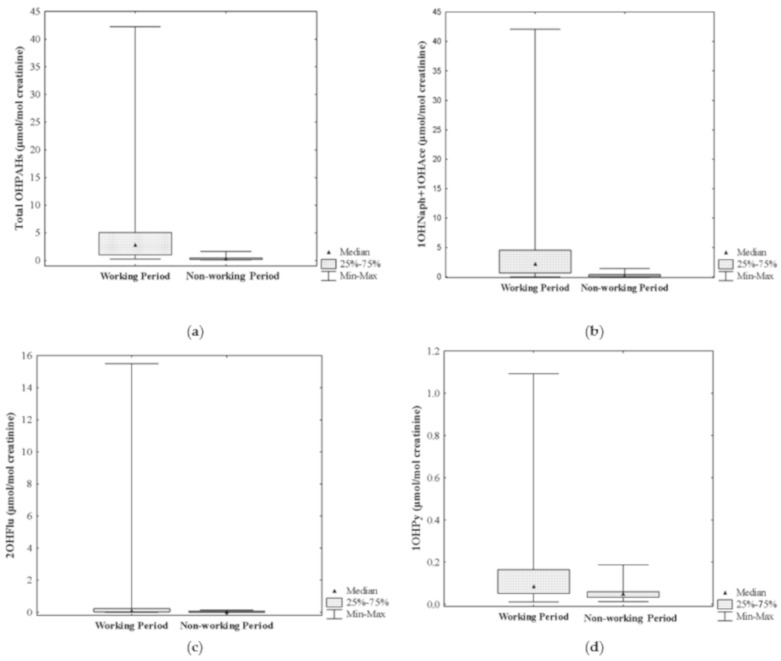

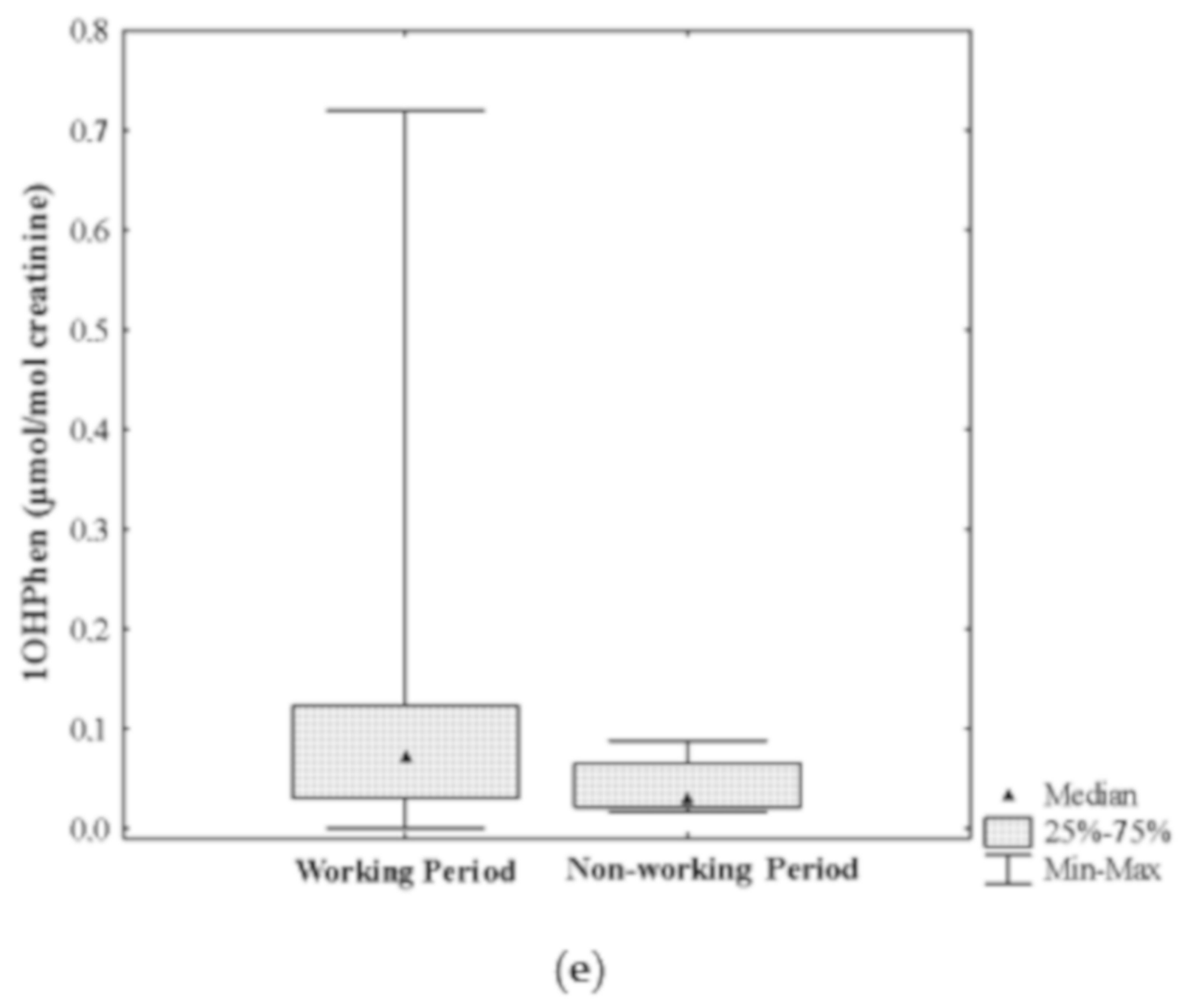
Urinary concentrations of PAHs biomarkers of exposure in grill workers during working and nonworking periods: (**a**) ∑OHPAHs, (**b**) 1-hydroxynaphthalene and 1-hydroxyacenaphthene, (**c**) 2-hydroxyfluorene, (**d**) 1-hydroxypyrene, and (**e**) 1-hydroxyphenanthrene. * Indicates statistical different medians (*p* ≤ 0.001) between each group of grill workers.

Urinary concentrations of ∑OHPAHs were significantly higher during a regular working period comparatively with the following resting days (ca. 9 times higher: 2.77 (0.213–42.3) versus 0.298 (0.097–1.66) µmol/mol creatinine, respectively; *p* ≤ 0.001) (Figure 2a). Moreover, levels of individuals compounds were also significantly higher during the regular working hours comparatively with the nonworking period (1OHNaph + 1OHAce: 2.23 (0.025–42.1) versus 0.098 (0.029–1.41) µmol/mol creatinine; 2OHFlu: 0.112 (8.49 × 10^−5^ − 15.5) versus 0.018 (1.24 × 10^−4^ − 0.133) µmol/mol creatinine; 1OHPy: 0.086 (0.011–1.09) versus 0.049 (0.013–0.188) µmol/mol creatinine; 1OHPhen: 0.073 (2.51 × 10^−4^ − 0.719) versus 0.031 (0.016–0.088) µmol/mol creatinine, respectively; *p* ≤ 0.001) (Figure 2b–e).

Regarding the individual biomarkers of exposure, the metabolites of naphthalene and acenaphthene (1OHNaph + 1OHAce) and fluorene (2OHFlu) presented the highest increments (2175% and 522%, respectively), followed by 1OHPhen (135%) and 1OHPy (76%) (*p* ≤ 0.001). Previously, Singh and coworkers [7,8] reported significantly increased levels of OHPAHs, with increments ranging between 161% for 1OHNaph and 934% for 1OHPy, in the urine of Indian kitchen workers who were directly involved in food preparation comparatively with a control group of workers (Table 2). Those authors [7] found 7 out of the determined 16 PAHs in air, namely, naphthalene, acenaphthene, fluorene, phenanthrene, pyrene, chrysene, and indeno[1,2,3-cd]pyrene, all at concentrations that surpassed 0.2 mg/m^3^, the permissible exposure level defined by the American Conference of Governmental Industrial Hygienists [44]. To our best knowledge, only this information exists concerning the occupational exposure to PAHs in kitchen workers through the determination of other urinary biomarkers of exposure than 1OHPy (Table 2).

Levels of urinary 1OHPy found during working hours (median of 0.086 µmol/mol creatinine; range: 0.011–1.09 µmol/mol creatinine) were significantly lower than the concentrations reported in the literature for other kitchen workers, namely, chefs, bakers, and assistant cooks (overall range of 1.25–9.7 µmol/mol creatinine, maximum values of 13.1 µmol/mol creatinine in Chinese frying cooks that used repeated frying oil [15]; 0.69–3.93 ng/mL creatinine corrected, maximum of 4.75 ng/mL creatinine corrected in the staff of a commercial Indian kitchen [7,8]) (Table 2). The higher levels reported for cooking workers may be a consequence of the mixed use of different cooking methods other than just grilling (e.g., deep frying, stir frying, steaming, stewing) in commercial kitchens [4,5,15,37]. Moreover, the great variability reported on the levels of urinary OHPAHs can also be promoted by the way authors express the urinary concentrations. Singh et al. [7,8] reported the urinary concentrations of individual OHPAHs as both mean and median values, which highlighted the great differences among the reported levels (e.g., mean of 3.93 versus median of 2.76 ng of 1OHPy/mL creatinine corrected in exposed kitchen staff from a commercial Indian kitchen). In addition, some PAH biomarkers of exposure were reported as not detected when expressed as median and with a quantified value when the concentrations were expressed as mean [7,8]. In the case of Portuguese restaurants, grillroom workers have a specific area for grilling the different foods, which is completely independent from the kitchen working area; no other cooking activities are performed by grill workers. In addition, the evaluated grillrooms’ working areas were all equipped with an exhaustion system that was regularly checked, which can help to explain the reduced Portuguese grill workers exposure to grilling emissions. Levels of urinary 1OHPy found in Portuguese grill workers were below the value of 0.5 µmol/mol creatinine, which is the proposed benchmark level for the occupational exposure to PAHs [44,45]. However, 5% of the collected samples from occupationally exposed grillroom workers presented urinary concentrations that exceeded that guideline (Figure 2d).

Urinary 3OHBaP is classified as the biomarker of exposure to carcinogenic PAHs and was only detected in 13% of participants and during their working hours. Furthermore, 3OHBaP data concerning the occupational exposure of restaurant workers are scarce (Table 2) with only one preliminary study reporting information. This study also reported a low detection rate of this metabolite in the occupationally exposed group [35]. Moreover, other studies also reported low detection rates of urinary 3OHBaP in firefighters immediately after their participation in firefighting activities, and in traffic policemen, and chimney-sweeps workers [26,46,47]. Overall, exposed grill workers presented 3OHBaP values that ranged between 0.98 and 2.67 nmol/mol creatinine (median of 1.71 nmol/mol creatinine), which are about 11 times higher than the concentrations previously reported (0.153 nmol/mol) (Table 2). Considering other occupationally exposed groups, urinary concentrations of 3OHBaP in exposed grill workers were also predominantly higher than the levels reported for metallurgy (0.02–0.74 nmol/mol creatinine) and aluminum electrode production plant (0.04–0.80 nmol/mol creatinine) workers [25,48].

### 3.3. Excretion Profiles of Urinary OHPAHs in Grill Workers

Excretion of OHPAHs from the human body is conditioned by the route of exposure (inhalation, ingestion, and dermal contact) and the molecular weight of the individual metabolite with evidences suggesting that lighter compounds are mostly eliminated through the urine, while the heaviest through the feces [47,49,50]. So far, information related with urinary excretion rates of OHPAHs and their pharmacokinetics is limited. Urinary 1OHPy has a half-life excretion rate ranging between 6 and 35 h and up to 13 h after inhalation and dermal contact exposures, respectively [51,52,53,54,55]. More recently, Li and coauthors [56] reported half-life times of urinary OHPAHs that successively increased according to the increased molecular weight of the respective PAH (1OHNaph—6.6 h; 2OHFlu—8.4 h; 1OHPhen—13.8 h; 1OHPy—23.5 h). Other authors also proved that different tasks performed by exposed workers strongly conditioned the urinary excretion rates of OHPAHs [48,50,57,58]. Moreover, Motorykin et al. [59] evaluated the excretion rates of 10 urinary OHPAHs after the consumption of smoked salmon by 9 members of the Confederated Tribes of the Umatilla Indian Reservation. Those authors found half-lives ranging between 1.7 h for 9-hydroxyfluorene and 7.0 h for 3-hydroxyfluorene, with concentrations returning to the background levels after 24 h after ingestion of smoked salmon. Therefore, more studies including the collection of spot urine samples before the working day starts, during working breaks, and at the end of the working day are needed to better understand the relation between metabolism and excretion rates of these compounds with the different routes of exposure to PAHs.

**Figure 3:**
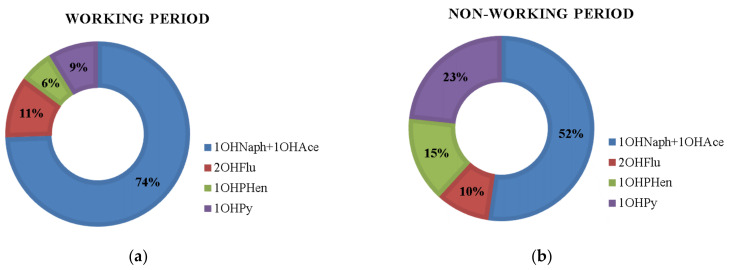
Representative profiles of urinary excretion of total PAH metabolites (∑OHPAHs) in grill workers from restaurants during regular working (**a**) and nonworking (**b**) periods.

Urinary concentrations of OHPAHs presented different profiles of distribution according to subjects’ working and resting periods: 1OHNaph + 1OHAce (74% of ∑OHPAHs) > 2OHFlu (11%) > 1OHPy (9%) > 1OHPHen (6%) in the days performing grilling activities and 1OHNaph + 1OHAce (52%) > 1OHPy (23%) > 1OHPHen (15%) > 2OHFlu (10%) during nonworking days (Figure 3).

**Figure 4:**
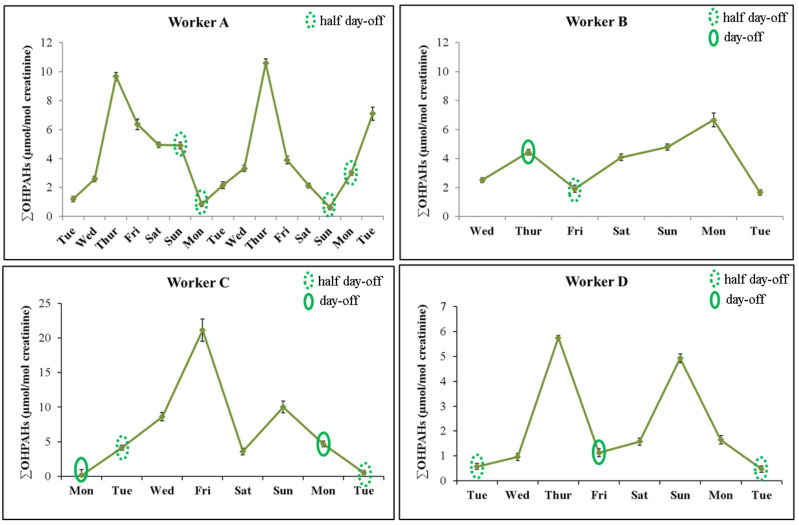
Urinary distribution profile of PAH biomarkers of exposure (1OHNaph + 1OHAce: 1-hydroxynaphthalene and 1-hydroxyacenaphthene; 2OHFlu: 2-hydroxyfluorene; 1OHPHen: 1-hydroxyphenanthrene; 1OHPy: 1-hydroxypyrene) in grill workers during working and nonworking periods.

The different distribution profiles of OHPAHs highlight the impact of abstention and/or participation in regular grilling activities. Figure 4 presents representative profiles of urinary excretion of ∑OHPAHs in the characterized workers during regular working and nonworking periods. Overall, levels of ∑OHPAHs presented a cumulative increase during the consecutive working days and predominantly decreased on the following resting period. Evidences were more pronounced in worker A who donated urine samples over 2 consecutive weeks. For that worker, concentrations of ∑OHPAHs were significantly increased during 3 consecutive days and then slightly decreased till the resting period (Figure 4). Grill workers from this restaurant never had a complete day-off during the sampling period, only two half-days off on Sunday and Monday (after 3 p.m.); also, this restaurant presented a much higher number of customers during the weekdays than in the weekend. Depending on the workflow during Sunday’ lunch time, levels of ∑OHPAHs presented a slight decrease on the first sampling week and a moderate increase on the second week. In the distribution profile of worker B, levels of ∑OHPAHs increased in the day-off comparatively with the previous working day, which can be attributed to an unusual high work and later end-shift (up to 2:00 a.m.) followed by the collection of the urine sample at the beginning of the following morning instead in the end of the resting day, as requested by the research team. Grillroom worker C had a resting period of a consecutive 1 and a half day, and as expected during that period the urinary concentrations of ∑OHPAHs slightly decreased after some working days (Figure 4). Participant D represents grill workers that do not have a consecutive day and a half of resting period. During a regular working week, this grillroom worker presented two peaks of urinary excretion of ∑OHPAHs, being both peaks observed after 2 consecutive working days and after a half-day or a complete day-off (Figure 4). In addition, the highest urinary excretion of OHPAHs was observed when only a half-day off was registered. The profile of urinary excretion observed for worker D clearly revealed the impact of resting period on grill workers’ occupational exposure. The urinary levels of OHPAHs need to be monitored in a higher number of grill workers during a prolonged period of work to better explore and validate these findings. In addition, a more complete data set (including grilling times, temperature, and kind of foods grilled) would allow a better characterization of the sources of exposure and the development of mitigations actions.

### 3.4. Urinary OHPAHs Correlations

Spearman correlations were calculated to assess the inter-relation among individual PAH biomarkers of exposure and ∑OHPAHs in the selected group of workers during working and nonworking periods (Table 3). Levels of ∑OHPAHs were positively correlated with the concentrations of 1OHNaph and 1OHAce during working (r = 0.967; *p* ≤ 0.01) and nonworking (r = 0.656; *p* ≤ 0.01) periods (Table 3). Previous studies reported naphthalene and/or acenaphthene as being among the most predominant PAHs in the breathable air of kitchen and food night market workers [7,30,34], thus highlighting the predominant contribution of inhalation to total exposure to PAHs. During working periods, levels of 2OHFlu were moderately correlated with the concentrations of other individual compounds (0.504 < r < 0.549; *p* ≤ 0.01), except with 1OHNaph + 1OHAce (Table 3). In addition, moderate associations were found among the urinary levels of 1OHPy and 1OHPhen (r = 0.564; *p* ≤ 0.01) during the working period of subjects. At the grillroom of the studied restaurants, there were two predominant sources of PAHs, the fumes from food processing and those released by the incomplete burning/pyrolysis of charcoal; no other cooking activities (e.g., frying) were performed during the sampling periods. The prevalence of these two major sources of PAHs can explain the moderate and even the low correlations found among the urinary levels of OHPAHs in occupationally exposed grill workers. A more complete identification and characterization of the predominant sources and routes of exposure to PAHs would be possible with the simultaneous assessment of PAHs in the breathing air zone and dermal exposed areas of grill workers and the urinary concentrations of their respective metabolites.

Regarding the resting period of grill workers, levels of urinary ∑OHPAHs were moderately correlated with the concentrations of 1OHNaph + 1OHAce, 2OHFlu, and 1OHPy (0.397 < r < 0.731; *p* ≤ 0.05) (Table 3). Concentrations of 1OHPy were well correlated with the excreted concentrations of 2OHFlu (r = 0.607; *p* ≤ 0.01) and 1OHPhen (r = 0.535; *p* ≤ 0.01).

**Figure 5:**
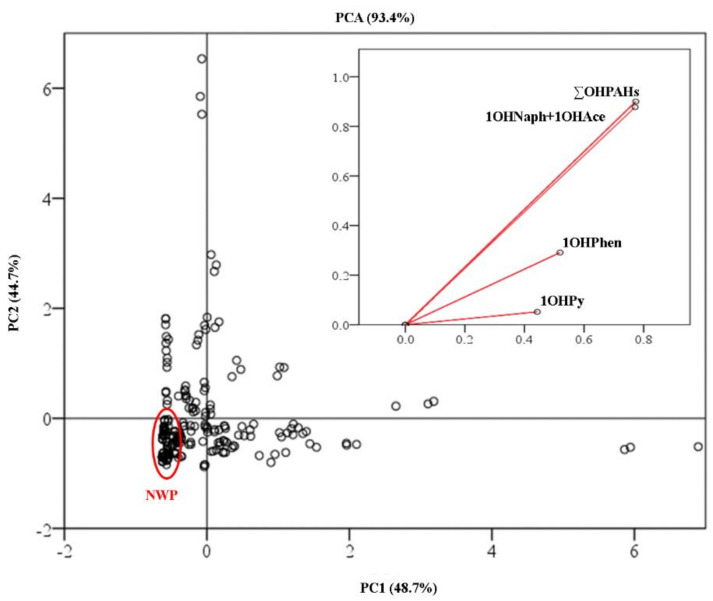
Principal components analysis constructed with the scores for each grill worker during working and nonworking (NWP) periods and based on the concentrations of urinary total (∑OHPAHs) and individual compounds (1OHNaph + 1OHAce: 1-hydroxynaphthalene and 1-hydroxyacenaphthene; 1OHPHen: 1-hydroxyphenanthrene; 1OHPy: 1-hydroxypyrene).

Principal component analysis (PCA) based on levels of OHPAHs was performed to evaluate if urinary levels of OHPAHs can be used as occupational exposure descriptors for grillroom workers (Figure 5). Two principal components were extracted from this PCA model, with eigenvalues ≥1.79 (PC1 and PC2) and an acceptable value for sampling adequacy (Kaiser–Meyer–Olkin ≥ 0.50). The PCA model was elaborated with the urinary concentrations of ∑OHPAHs, 1OHNaph + 1OHAce, 1OHPhen, and 1HOPy and altogether both PC1 and PC2 represented 93.4% of the original data (Figure 5). Communalities of all the extracted components ranged between 0.898 and 0.971. PC1 explained up to 48.7% of the total variance with the levels of ∑OHPAHs, 1OHNaph, and 1OHAce presenting the highest loadings (component scores ≥ 0.965). The first function allowed a partial separation between grill workers during their working period from the resting period (Figure 4). PC2 represented 44.7% of the original data and was strongly loaded by the urinary concentrations of 1OHPhen and 1OHPy (component scores ≥0.924), which contributed to a better differentiation among the two groups (Figure 5). This PCA model suggests that urinary levels of OHPAHs can be used as predictor of grillroom workers’ occupational exposure to PAHs. These findings are in line with the predominantly increased concentrations of total and individual OHPAHs found in the urine of grill workers during working periods comparatively with nonworking periods (Figure 2). However, other determinants of exposure such as the number of working hours, the food grilled, and the type of grill and ventilation used should be evaluated in future studies. Despite the limited information available, some evidences also point to the use of 1OHPy as a determinant predictor of increased concentrations of different biomarkers of oxidative stress, namely, malondialdehyde, 8-hydroxy-2-deoxyguanosine, and isoprostane, in the urine of kitchen workers [15,29,37]. Wang et al. [37] reported increased concentrations of 1OHPy, 8-hydroxy-2-deoxyguanosine, and malondialdehyde in the urine and higher levels of binucleated micronucleus frequency, and comet tail length in the serum of exposed kitchen workers comparatively to a control group. Moreover, Wang et al. [37] proved that greater concentrations of those biomarkers were directly associated with the number of years as restaurant’ kitchen workers and with the daily time spent with cooking activities. Other authors reported the association between increased levels of 1OHPy with a significant decline in lung function and kidney injury among kitchen workers in comparison with a control group [8,60]. Occupational exposures to cooking emissions, principally cooking oil fumes, have been also related with an increased predisposition to develop lung cancer [14,61,62]. More comprehensive studies including grill workers’ occupational exposure to PAHs and their relationship with urinary biomarkers of exposure and/or of effect are needed to investigate the potential risks for human health.

## 4. Conclusions

Since information on the topic is almost inexistent, this study assessed the levels of six PAHs biomarkers of exposure in the urine of grill workers’ during a regular working period. Concentrations of total OHPAHs were significantly increased during subjects’ working period comparatively with the following resting days (median of 2.77 versus 0.298 µmol/mol creatinine; ca. nine times higher), being 1OHNaph + 1OHAce (median 2.23 versus 0.098 µmol/mol creatinine) and 2OHFlu (median 0.112 versus 0.018 µmol/mol creatinine) the compounds that increased the most (ca. 23- and 6-fold increase, respectively). The lowest increments (0.086 versus 0.049 µmol/mol creatinine; about 2 times higher) were found for 1OHPy, however, about 5% of the collected samples exhibited urinary 1OHPy levels that were higher than the recommended benchmark value [44]. Thus, these results suggest that other OHPAHs should be monitored besides 1OHPy to have a more comprehensive assessment of the real total exposure of exposed workers. 3OHBaP was only detected in a small group of exposed grillroom workers. This work demonstrate that grill workers are at high risk of exposure to PAHs and evidences suggests that individual and cumulative concentrations of ∑OHPAHs can be used as predictors of occupational exposure to grilling activities. However, more studies including a higher number of participants being monitored over a longer period of time and addressing different determinants of exposure are needed to explore the achieved findings and to promote control safety measures to reduce the occupational exposure of grill workers. Moreover, monitorization of workers’ breathing air should be also considered to explore the relation between airborne and/or dermal PAHs and its possible sources with the urinary levels of monohydroxyl-PAHs, which will pursue a more complete health risk assessment.

## Figures and Tables

**Table 1:** Characterization of grill workers (*n* = 18).

**Personal characteristics**	
Age (mean ± SD; years)	35.7 ± 9.8
Weight (mean ± SD; kg)	85.0 ± 9.1
Body mass index (mean ± SD; kg/m^2^)	28.3 ± 4.5
**Work experience**	
Working days per week (mean ± SD; days)	5.5 ± 0.5
Working hours per day (mean ± SD; hours)	9.6 ± 2.8
Working period performing grilling activities (mean ± SD; hours)	4.6 ± 2.2

**Table 2:** Concentrations of polycyclic aromatic hydrocarbon (PAH) metabolites (mean ± SD or 95% confidence intervals; µmol/mol creatinine unless indicated otherwise) in the urine of restaurant workers.

Continent, Country (City)	Study Population	*n*	Age	Notes	Urinary Metabolite ^a^	Reference
Europe, Portugal (Amarante, Maia, Matosinhos, Porto, Valongo, Vila Nova Gaia)	Grill workers	18	(20–48)	Working period	1OHNaph + 1OHAce: 2.23	This study ^#^
					2OHFlu: 0.112	
					1OHPy: 0.086	
					1OHPhen: 0.073	
				Nonworking period	1OHNaph + 1OHAce: 0.098	
					2OHFlu: 0.018	
					1OHPy: 0.049	
					1OHPhen: 0.03	
Europe, Portugal	Grill workers	n.r.	(31–35)	Exposed	1OHPy: 0.068	[35] ^#^
(Porto)					3OHBaP: 0.153 × 10^−3^	
				Nonexposed	1OHPy: 0.050	
					3OHBaP: 0.127 × 10^−3^	
Asia, China (Taiwan)	Restaurant workers	93	39.3 ± 11	Kitchen staff	1OHPy: 6.0 ± 8.0	[4]
		61	35.6 ± 12.5	Service staff	1OHPy: 2.4 ± 4.3	
Asia, China	Restaurant workers	202	42.5 ± 9.7	Kitchen staff	1OHPy: 2.33 ± 2.43	[5] *
(Taiwan)		185	40.4 ±12.7	Service staff	1OHPy: 1.40 ± 2.54	
Asia, China	Restaurant workers	67	29.0 ± 5.6	Exposed group	1OHPy: 1.25 (0.69–1.71)	[37] ^#^
(Guangzhou)		43	27.6 ± 7.5	Control group	1OHPy: 0.83 (0.61–1.07)	
Asia, China	Military services	61	21.4 ±1.8	Military cooks		[29] **
(Taiwan)				Preshift	1OHPy: 0.46 (0.25–0.66)	
				Postshift	1OHPy: 0.69 (0.25–1.12)	
		37	23.1 ± 2.4	Administratives		
				Preshift	1OHPy: 0.36 (0.18–0.53)	
				Postshift	1OHPy: 0.20 (0.10–0.31)	
Asia, China (Shenzhen)	Restaurant workers	63	26.2 ± 6.0	Deep-frying cooks	1OHPy: 3.8 (2.8–5.6)	[15]
		55	24.4 ± 5.2	Frying cooks ^b^	1OHPy: 9.7 (6.4–13.1)	
		51	26.7 ± 6.5	Frying cooks ^c^	1OHPy: 4.4 (3.0–6.2)	
		67	24.8 ± 4.9	Service staff	1OHPy: 2.5 (1.6–3.7)	
Asia, India (Lucknow)	Kitchen workers	94	32.0 ± 8.3	Kitchen staff	1OHNaph: 10.69 (8.37–13.01)	[7,8] **
					2OHFlu: 3.55 (2.60–4.51)	
					3OHFlu: 2.60 (1.90–3.32)	
					9OHFlu: 1.44 (1.0–1.89)	
					9OHPhen: 0.98 (0.68–1.27)	
					1OHPy: 3.93 (3.11–4.75)	
		94	31.7 ± 9.4	Control group	1OHNaph: 4.10 (0.36–7.85)	
					2OHFlu: 1.22 (0.30–2.13)	
					3OHFlu: 0.83 (0.19–1.47)	
					9OHFlu: 0.36 (0–0.97)	
					9OHPhen: 0.29 (0–0.79)	
					1OHPy: 0.38 (0–1.46)	
Asia, Thailand	Street vendors	14	(15–40)	Grilled-meat	1OHPy: 0.15	[36] ^#^
(Bangkok)				Control group	1OHPy: 0.04	

^a^ 1OHPy: 1-hydroxypyrene; 3OHBaP: 3-hydroxybenzo(a)pyrene; 1OHNaph: 1-hydroxynaphtahlene; 1OHAce: 1-hydroxyacenaphthene; 2OHFlu: 2-hydroxyfluorene; 3OHFlu: 3-hydroxyfluorene; 9OHFlu: 9-hydroxyfluorene; 9OHPhen: 9-hydroxyphenanthrene. ^b^ repeated frying oil; ^c^ restaurant waste oil; * Data were converted to µmol/mol creatinine by dividing the reported values/1.93. ** Concentrations are expressed in ng/mL creatinine corrected; ^#^ Concentrations are expressed as median and/or inter-quartile range; n.r.—not reported.

**Table 3:** Correlation matrix between the concentrations of urinary PAH metabolites in grill workers during their working and nonworking periods.

								R^2^
PAH biomarkers of exposure *		2OHFlu	1OHPhen	1OHPy	∑OHPAHs			1
**1OHNaph + 1OHAce**	Working period							0.9
	Non-working period							0.8
**2OHFlu**	Working period							0.7
	Non-working period							0.6
**1OHPhen**	Working period							0.5
	Non-working period							0.4
**1OHPy**	Working period							0.2–0.3
	Non-working period							≤0.2

* 1OHNaph + 1OHAce: 1-hydroxynaphthalene and 1-hydroxyacenaphthene; 2OHFlu: 2-hydroxyfluorene; 1OHPHen: 1-hydroxyphenanthrene; 1OHPy: 1-hydroxypyrene; 3OHBaP: 3-hydroxybenzo(a)pyrene; ∑OHPAHs: total PAH metabolites.

## Data Availability

No new data were created or analyzed in this study. Data sharing is not applicable to this article.

## Supplementary Materials

The following are available online at https://www.mdpi.com/1660-4601/18/1/230/s1, Table S1: Calibration data obtained (*n* ≥ 6) for the analyzed polycyclic aromatic hydrocarbon (PAH) metabolites in the urine of grill workers. Figure S1: High Performance Liquid Chromatography with fluorescence detection chromatograms of solvent baseline (pink line), standard mixture containing 6 PAH metabolites (1OHNaph+1OHAce—80.80 mg/L, 2OHFlu—0.40 μg/L, 1OHPhen—0.40 μg/L, 1OHPy—0.40 μg/L, 3OHB(a)P—0.40 μg/L) (black line), a grill worker urine sample during non-working period (green line) and a grill worker urine sample during working period (blue line).
