# Supplementary material for: Grill Workers Exposure to Polycyclic Aromatic Hydrocarbons: Levels and Excretion Profiles of the Urinary Biomarkers
Source: Int J Environ Res Public Health. 2020 Dec 30;18(1):230. doi: 10.3390/ijerph18010230 (PMC7796024; doi:10.3390/ijerph18010230)
Supplement: Supplementary file 1 [file ijerph-18-00230-s001.pdf]

International Journal of
Environmental Research and Public Health

MDPI

Supplementary Material

Grill Workers Exposure to Polycyclic Aromatic Hydrocarbons: Levels and Excretion Profiles of the Urinary Biomarkers

Marta Oliveira, Sílvia Capelas, Cristina Delerue-Matos and Simone Morais

Table S1. Calibration data obtained (n ≥ 6) for the analysed PAH metabolites in the urine of grill workers.

Compound[a]	Calibration range	Regression equation[b]	R^2	LOD[c]	LOQ[c]
1OHNaph+1OHAce (mg/L)	4.04-80.80	y = 9484 x - 26589	0.9996	0.195	0.650
2OHFlu (μg/L)	0.020-0.250	y = 618137 x - 2554	0.9991	0.800 $\times10^{-3}$	2.80$\times10^{-3}$
1OHPhen (μg/L)	0.060-0.500	y = 363043 x - 14797	0.9991	2.20 $\times10^{-3}$	7.30 $\times10^{-3}$
1OHPy (μg/L)	0.060-0.500	y = 396248 x - 7289	0.9990	2.60 $\times10^{-3}$	8.50 $\times10^{-3}$
3OHBaP (μg/L)	0.020-0.400	y = 918840 x - 9401	0.9990	1.10 $\times10^{-3}$	3.50 $\times10^{-3}$

[a] 1OHNaph: 1-hydroxynaphthalene; 1OHAce: 1-hydroxyacenaphthene; 2OHFlu: 2-hydroxyfluorene; 1OHPHen: 1-hydroxyphenanthrene; 1OHPy: 1-hydroxypyrene; 3OHBaP: 3-hydroxybenz(a)pyrene; [b] y - absorbance; x - concentration (mg/L); [c] Data is presented on urine basis by applying a conversion factor of 0.10 to the LOD and LOQ on a solvent basis.

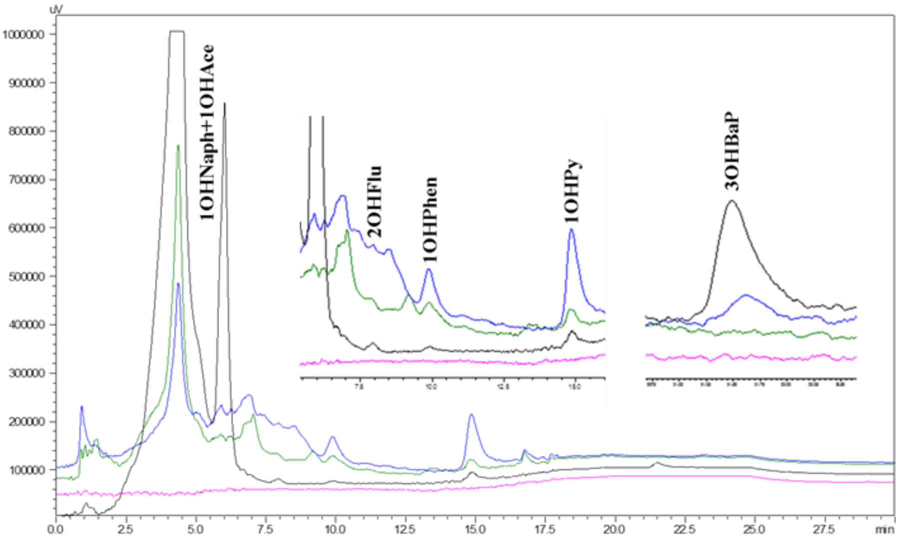

Figure S1. High Performance Liquid Chromatography with fluorescence detection chromatograms of solvent baseline (pink line), standard mixture containing 6 PAH metabolites (1OHNaph+1OHAce – 80.80 mg/L, 2OHFlu – 0.40 μg/L, 1OHPhen – 0.40 μg/L, 1OHPy – 0.40 μg/L, 3OHB(a)P – 0.40 μg/L) (black line), a grill worker urine sample during non-working period (green line) and a grill worker urine sample during working period (blue line).
